# Using a business model approach and marketing techniques for recruitment to clinical trials

**DOI:** 10.1186/1745-6215-12-74

**Published:** 2011-03-11

**Authors:** Alison M McDonald, Shaun Treweek, Haleema Shakur, Caroline Free, Rosemary Knight, Chris Speed, Marion K Campbell

**Affiliations:** 1 Health Services Research Unit, University of Aberdeen, Health Sciences Building, Foresterhill, Aberdeen, UK, AB25 2ZD; 2 Division of Clinical and Population Sciences and Education, University of Dundee, Dundee, UK, DD2 4BF; 3 London School of Hygiene and Tropical Medicine, London, UK, WC1E 7HT; 4 Newcastle Clinical Trials Unit, Newcastle Upon Tyne, UK, NE2 4HH

## Abstract

Randomised controlled trials (RCTs) are generally regarded as the gold standard for evaluating health care interventions. The level of uncertainty around a trial's estimate of effect is, however, frequently linked to how successful the trial has been in recruiting and retaining participants. As recruitment is often slower or more difficult than expected, with many trials failing to reach their target sample size within the timescale and funding originally envisaged, the results are often less reliable than they could have been. The high number of trials that require an extension to the recruitment period in order to reach the required sample size potentially delays the introduction of more effective therapies into routine clinical practice. Moreover, it may result in less research being undertaken as resources are redirected to extending existing trials rather than funding additional studies.

Poor recruitment to publicly-funded RCTs has been much debated but there remains remarkably little clear evidence as to why many trials fail to recruit well, which recruitment methods work, in which populations and settings and for what type of intervention. One proposed solution to improving recruitment and retention is to adopt methodology from the business world to inform and structure trial management techniques.

We review what is known about interventions to improve recruitment to trials. We describe a proposed business approach to trials and discuss the implementation of using a business model, using insights gained from three case studies.

## Introduction

Randomised controlled trials (RCTs) are widely accepted as the gold standard for evaluating health care interventions [1,2]. Several factors contribute to the success of a RCT, including a research question that is relevant to those at whom the trial results are aimed [3,4], a design that is both scientifically rigorous and which fits in with clinical practice [5], obtaining the appropriate legislative approvals [6], the active participation and commitment [7] of clinical sites/practices, appropriate analysis and/or reporting and of course the willingness of individuals to take part. Each of these stages can individually or collectively be barriers to the successful delivery of a trial.

One of the most common problems, however, is with recruitment. Many studies fail to meet their recruitment targets, or fail to meet them without extending the length of the trials. McDonald et al [8], for example, found that of 114 trials, only 38 (31%) achieved their original recruitment target and 65 (53%) were extended.

The reasons why certain trials recruit well while others do not remain unclear [5]. Several potential limiting factors have been identified in the literature including constraints on clinician time [9.10], lack of available staff [11], impact on clinician autonomy, complexity of trial procedures [12], overestimating the number of patients available for study participation [13] and the perceived relevance of the research question to the clinicians [4]. Barriers to patients' involvement include lack of knowledge and trust in trials and unacceptability of randomisation [14].

A recent systematic review of interventions to improve recruitment to randomised controlled trials identified 27 eligible healthcare trials, including over 26,604 participants [15]. There were 24 studies involving interventions aimed directly at trial participants, while three evaluated interventions aimed at those recruiting participants. Many studies looked at recruitment to hypothetical trials and it is unclear how applicable these results are to real trials. Some interventions were effective in increasing recruitment: telephone reminders to non-respondents following a written invitation to take part in a trial (RR 2.66 95% CI 1.37 to 5.18), use of opt-out, rather than opt-in, procedures for contacting potential trial participants (RR 1.39 95% CI 1.06 to 1.84) and open designs where participants know which treatment they are receiving in the trial (RR1.25 95% CI 1.18 to 1.34).

An earlier systematic review with slightly different inclusion criteria [16] came to similar conclusions, although it also reported monetary incentives and culturally sensitive trial materials to be effective. Both reviews found the literature on the effect of recruitment interventions to be both sparse and often of poor methodological quality.

There are a number of proposals to increase recruitment to trials. Campbell et al [17] agreed that, to be successful, trialists should base their design on respecting the needs of patients and clinical professionals. Sackett [7] also described how, in multi-centre RCTs, a responsibility of each site should be to involve and train those who will be recruiting and following-up trial participants. He stated that such action would enable collaborators to 'buy-in' to the trial and develop both ownership and commitment, terms more familiar in the business world than in health research.

One proposed solution to improving recruitment and retention is to adopt methodology from the business world to inform and structure trial management techniques [18]. Francis et al [18] developed a reference model (developed from marketing theory), which could be used for improving trial processes and as such, potentially improve recruitment to trials. However, whether this approach is useful in practice remains unclear.

This paper, therefore, seeks to a) describe the proposed business approach to trials and b) discuss the implementation of a business approach using insights gained from three case studies.

### A business approach

**Figure 1:**
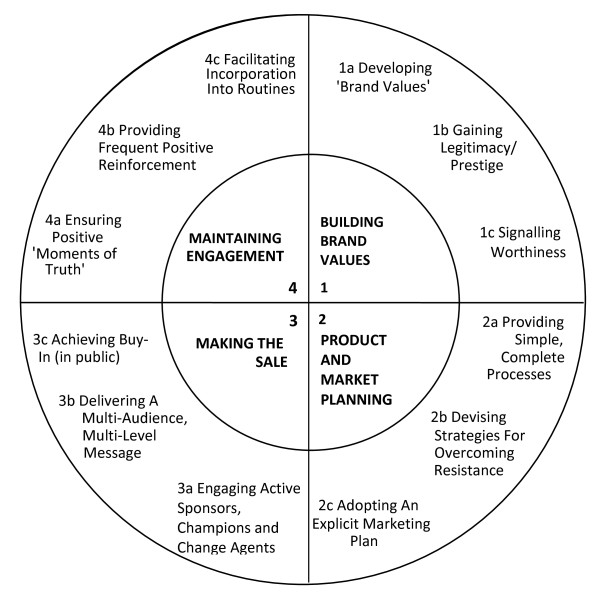
**The business model**. Illustrates the four domains of the model and the three components of each domain (from Francis et al [18]).

Francis et al [18] first discussed whether clinical trials could be regarded as businesses and suggested that dimensions of running a successful trial include 'marketing', 'sales' and 'ongoing client management' which require a range of effective management techniques paralleling those for running a successful business. Francis et al used multiple methods to develop a reference model using insights from marketing theory, which could be used for ongoing assessment of the sales and marketing capability of a trial. The model has four domains: (1) Building Brand Values (2) Product and Market Planning (3) Making the Sale and (4) Maintaining Engagement. Each of the four domains has three components (see Figure 1). The twelve components are considered as links in a chain; if one link is underdeveloped then the whole chain is weakened. Table 1 includes our translation of the model into language that trialists are more familiar with.

**Table 1:** Translation of business model components

Component	Component description	How this translates for trialists
1a. Developing brand values	'Brand values' define what a 'brand 'is' and what it 'is not' - i.e. its 'personality'.	The 'brand' needs to convey clearly what a trial 'stands for'; what collaborators and participants can expect as being part of your study.

1b. Gaining legitimacy and prestige	Trials need legitimacy - they need to be positively 'tagged' by association with prestigious individuals and institutions.	At every stage in the trial demonstrate that the trial is being professionally managed. The support of respected funding bodies, Universities, clinical networks, academics and clinicians all contribute to this component.

1c. Signalling worthiness	It is vital to signal to collaborators and participants that this trial will create greater value than the costs (time and money) involved.	Demonstrate that the research question is extremely important. Can the trial be supported at recruiting sites by networks and/or research money? What benefits may participation and outcome bring to knowledge of the best treatments?

2a. Providing simple, complete processes	Trials require clinical staff to undertake work that is additional to their normal duties.	Getting a trial set-up, identifying, and recruiting participants, delivering the trial intervention and follow-up of participants all require effort. Data collection should be the minimum required and data entry/transfer processes should be as simple as possible. Clarity re roles and responsibilities is vital.

2b. Devising strategies for overcoming resistance	Potential participants frequently raise objections.	Recognise what anxieties collaborators are likely to have and develop accurate, standard responses. Ensure that the science of the trial is clear and unambiguous.

2c. Adopting an explicit marketing plan	The marketing of a trial is too important to be done informally. A formal marketing plan is required and should include a definition of target market segments (groups that need to buy in to the trial) and the trial's unique selling points (USPs).	Consider who the gatekeepers are and who should be approached to investigate participating in a study; consider why involvement in this trial is unique and the importance of answering the research question.

3a. Engaging active sponsors, champions and change agents	'Selling' a trial to prospective participants requires persuasion. This requires enrolling sponsors (public advocates), champions (activists) and change agents (facilitators).	Obtain and maintain the support of multiple groups such as disease specific networks, local experts in the disease area (e.g. local lead clinicians who can promote the study in their area), patient representatives and trial managers. Involve local investigators in promoting the study by giving presentations about the trial.

3b. Delivering a multi-audience, multi-level message	Trials need to convey sales messages through publicity, presentations, training materials, etc.	Communicate in the language of the persons being targeted (e.g. surgeons are more likely to be persuaded by different messages to administrators or nursing staff). Correct any misunderstandings by inviting feedback from audiences that the trial has been presented to and adapting the information as appropriate.

3c. Achieving buy-in (in public)	Public buy-in requires intended participants to announce their commitment to join the trial. When someone states, in public, that they are willing to undertake an action, then they are much more likely to do it.	Through websites, newsletters and other communication means, create and regularly update information on participating collaborators. Ensure that local teams are aware of research going on that may involve patients they deal with (e.g. ward staff). The local clinical leads should inform colleagues and team members of their participation in the trial enthusiastically and in person.

4a. Ensuring positive 'moments of truth'	People judge organisations on the basis of their experience at 'moments of truth'.	Have systems in place (e.g. dedicated email accounts, frequently asked questions on the trial website, prompt response by the trial manager to queries) to ensure all communications are handled efficiently and honestly. Providing competent and honest responses will increase loyalty to the trial and the research team and will result in a greater chance of ongoing successful collaborations.

4b. Providing frequent positive reinforcement	Positive reinforcement for existing participants should be an important part of a trial's 'participant retention strategy'. It is more 'expensive' to recruit new participants than to retain existing participants.	Provide regular updates of information and recognise successes. Keep participants informed e.g. through regular newsletters, website updates etc and sending small tokens of appreciation.

4c. Facilitating incorporation into routines	Activities that become embedded as routines are more likely to be done than 'one-offs'.	As far as possible, ensure the trial procedures are simple, in order that the participating sites can incorporate the study into their established routines. Flexibility may be required as processes can vary at different clinical sites.

Francis et al proposed that the model could be used in a variety of ways to help the conduct of clinical trials. These included: (1) to guide development of a recruitment plan, (2) as a diagnostic tool if trials have difficulties (and hence as a basis for deciding what type of remedial action to take), and (3) for auditing the progress of trials (to enable early identification of weaker managerial components and allow initiatives to strengthen them to be developed). As the model was developed from a theory-building process and only from a single trial, it was proposed as a tentative framework, and thus evidence is required from a range of trials to validate its findings.

To that end, we present below three case studies where the model was applied (these case studies represent trials known to the authors that had used the model). Each case study represents a slightly different use of the model, highlighting its versatility in the trials arena.

### Implementation of the business model

#### Case study 1: the CRASH-2 trial (using the model to develop trial processes)

CRASH-2 is a large multinational randomised placebo controlled trial among trauma patients with, or at risk of, significant haemorrhage, to investigate the effects of antifibrinolytic treatment on death and transfusion requirement [19]. Recruitment commenced in May 2005 and was completed successfully in January 2010. In the set up phase of this trial, which aimed to recruit 20,000 trauma patients within 4.5 years, the business model developed by Francis et al [18] was applied to plan the overall trial management. Below we illustrate in detail how the twelve components of the model were used to guide the management of the trial.

### Developing brand values

The 'CRASH' brand was already established with some patient organisations and several hundred emergency and critical care doctors worldwide following the successful delivery of the CRASH trial [20]. At the end of the CRASH trial 50 key collaborators were asked about the reasons they took part in the trial and what aspects of the trial coordinating centre's performance they would like to see improved. This information allowed the CRASH-2 coordinating team (the same team as ran the CRASH trial) to identify both the positive aspects and weaknesses of the CRASH 'brand'. Overall, the collaborators viewed their association with the CRASH trial as a very positive and worthwhile experience. Therefore, continued use of the original 'CRASH' theme in the logo and acronym for the CRASH-2 trial was considered useful to build on the positive image already established.

### Gaining legitimacy and prestige

Factors which were identified by CRASH trial collaborators as giving the trial legitimacy and prestige were enshrined into the CRASH-2 trial. These factors included trial coordination by an academic institution, funding by a non-commercial organisation and management and overview by respected individuals in the relevant clinical and academic fields. Obtaining approval from all relevant ethics and regulatory agencies is required legally but this was also important for assuring collaborators about the ethical aspects of the trial and its compliance with relevant local legal requirements. In addition, approval for the trial was obtained from the World Health Organisation Ethics Committee as this was considered a positive 'tag' from a relevant prestigious organisation when marketing the trial globally. It was important to consider who the collaborating investigators viewed as 'prestigious' individuals and institutions' and engagement of these was maintained throughout the course of the trial.

### Signalling worthiness

Recognising that even the simplest of trials would increase the workload for clinicians and their teams, the CRASH-2 trial utilised a simple pragmatic design and the trial-related procedures were kept to a minimum. CRASH-2 also emphasised the values that would appeal to individual collaborators, such as the importance of the trial question, contribution of the trial to advancing medical knowledge, how the trial might potentially influence patients' chances of survival in the future, positive aspects of being part of a global network with a common focus, potential for authorship or being named in the publication, potential to improve career prospects through being part of a research collaboration and 'hands-on' experience on how a trial is organised and conducted.

### Providing simple, complete processes

The CRASH-2 trial identified and minimised those tasks which could only be done by the collaborators at recruiting sites (recruitment and follow-up of participants) and maximised those which could be done by the trial coordinating centre (TCC). For example, the TCC took responsibility for completing and assembling documents for ethics and regulatory application, liaising with ethics committees as much as possible, responding to any comments made and ensuring that reporting requirements were fulfilled. Randomisation methods also took into consideration local needs. For example, if international telephone lines were not available, an alternative randomisation method was made available. Multiple methods for data collection were also implemented taking into consideration the availability of fax machines, internet connection and computer software. Collaborators could choose a data collection method most suitable for their situation. All guidance on trial processes were made simple, translated into local languages where required and were easily accessible in the site study file and on the trial collaborators website. Without simplicity and complete processes, the trial team recognised that buy in was likely to be lower and the possibility of dropout from the trial would be increased.

### Strategies for overcoming resistance

Factors were identified that might cause a potential collaborator to reject the CRASH-2 trial or undertake it with less than full commitment; for example colleagues not being supportive of the trial, anxieties about putting patients into a research trial, anxieties about how to explain the trial to relatives, the amount of paperwork involved, the lack of incentives for junior staff to recruit and not remembering to recruit. In a concerted attempt to address these issues directly, the TCC made training presentations and responses to frequently asked questions available to collaborators.

### Adopting an explicit marketing plan

A formal marketing plan was developed that included a definition of target market segments i.e. groups that needed to buy-in to the trial. The CRASH-2 trial needed to engage a wide variety of people and organisations including funders, academics, clinicians, nurses, administrators, ethics committees, regulatory agencies, participants and their relatives, patient organisations, academic press and drug manufacturers. Each person/group had varying levels of interest but involvement by each was required for the success of the trial. The uninformed needed to be told about the trial and this was done primarily by providing simple information using a variety of means including personal contacts, email, post, journal articles and advertising in the scientific press such as the Lancet website. Those who still had concerns after reading the information were contacted personally by someone from the TCC or another individual likely to be viewed as having legitimacy and prestige. Those who were interested but slow to act were supported and encouraged by the TCC and other collaborators (for example a local national coordinator). Collaborators who were recruiting well were highlighted in, for example, trial newsletters and on the trial website. A number of key collaborators were identified as being vital for the success of the trial. For CRASH-2, they were the ones who demonstrated that they could recruit a large number of the patient population required for the trial and were able to ensure that data collection was of the highest quality. Many of these were engaged as national coordinators for their countries and assisted in engaging others to join the collaboration. They shared their experience of the trial and its processes so others could learn from them.

### Engaging active sponsors, champions and change agents

In addition to clinical trialists understanding that a sponsor is the person/institution taking responsibility for the initiation, management and financing (or arranging the financing) of a trial [21], sponsors can also be considered as public advocates, champions as activists and change agents as facilitators of research [18]. It is also known that persuasion is more likely to occur if the advocate is respected and known personally to the prospective participant. For CRASH-2, this involved engaging and maintaining the support of multiple groups such as disease specific networks or societies, experts in the disease area (e.g. local lead clinicians who can promote the study in their area), patients and their representatives. As an international trial, engaging people within a country was required to ensure the message was relevant to that country and sensitive to their needs.

### Delivering a multi-audience, multi-level message

For the CRASH-2 trial, *multi-audience *referred to the different interest groups involved and *multi-level *referred to that which "would appeal to the heart and the head". CRASH-2 needed to communicate in the language of the persons being targeted. For example, doctors in general needed to be convinced by the science of the trial and information on the scientific evidence to support the conduct of the trial was crucial. However, many nurses wanted to know more about the potential benefits for patients to convince them to collaborate. Although the same information was being presented, at collaborators meetings for example, the key messages needed to use language which addressed both sets of concerns.

### Achieving buy-in (in public)

The trial team recognised that the trial impacted on more people at each site than solely the trial team. As such, the TCC advised local collaborators to ensure that local teams not directly involved in the trial, but who may be involved in the care of a trial participant, were aware of the trial, for example physiotherapists and pharmacists. To ensure that there was commitment to the trial, where possible (for example at collaborators meetings), collaborators were asked to state their commitment at the meeting because once someone states in public that they are willing to undertake an action, they are much more likely to actually do it [18].

### Ensuring positive 'moments of truth'

When collaborators needed to make contact, the CRASH-2 team attempted to make all dealings with the TCC positive. To enable this, the CRASH-2 trial developed systems such as dedicated email accounts, frequently asked questions (FAQs) on the trial website, ensured prompt response to queries and developed in-house procedures to ensure all communications were handled efficiently within set time frames. However, there were times when TCC procedures failed and in those circumstances an honest response, with an apology and a clear plan to resolve the problem, was given. The TCC believed that this was likely to be viewed more positively than trying to pretend nothing was wrong.

### Providing frequent positive reinforcement

Many of the doctors and nurses in the local trial team were involved in recruiting patients and completing the necessary paperwork for the trial. Recognising and rewarding these acts were seen as vital for them to continue to be motivated to deliver these tasks. Recognition included issuing certificates of participation, sending small tokens from the TCC after they recruited each patient and sending personalised text messages to thank them for their work. Also opportunities for networking, social interaction and the sharing of experiences at collaborators meetings were necessary.

### Facilitating incorporation into routines

CRASH-2 attempted to incorporate as many trial procedures as possible within local routine. For example, trial participants generally needed to have cross-matching for blood transfusions; attaching a CRASH-2 trial label/logo to all blood transfusion forms assisted the doctors/nurses to remember the trial as part of their standard care routine.

#### Case study 2: the TXT2STOP trial (using the model as a diagnostic tool)

TXT2STOP is a trial of smoking cessation support via text (sms) messages http://www.txt2stop.org/. The trial had been informed by a pilot study which had successfully recruited 200 participants in 17 days. By the time of recruitment to the full trial, however, the external environment had changed dramatically with the introduction of the "no-smoking in public places" policy introduced in the UK and concurrent large-scale stop smoking multi-media campaigns being run by the UK National Health Service (NHS). This resulted in only 1056 participants being recruited to the trial in the first eight and a half months (instead of the expected 2000 participants). At this time (June 2008), the business model was used as a framework for understanding approaches to increasing trial recruitment.

The model resulted in a new conceptualisation of recruitment. According to the model the trial was being promoted to three distinct groups (market segments) - directly to smokers, primary care teams and smoking cessation services. The trial team carried out a review of the existing literature and spoke with members of each group to identify what people in each of the selected market segments would "value" (ie what would encourage them to 'sign-up') and to identify anything that put them off joining or recruiting to the trial. Text messages and letters to potential participants were developed focussing on the potential benefits of participation and the factors that have been reported to encourage participants to join trials [22-27]. To ensure that the benefits of involvement were not outweighed by costs to the GPs, appropriate funding was secured via service support costs (attributed to the UK NHS) [28]. Letters were written directly to smokers on GP lists so that the trial was not reliant on GP or nurse clinical time telling patients about the study. The value of the trial was endorsed by stating University links in the letters. The trial team engaged active sponsors and the trial was promoted on the NHS smoking cessation websites in England, Wales and Scotland. All promotion activities and adverts were monitored to evaluate their time and monetary costs and effectiveness. Cost-effective strategies were repeated and less effective strategies abandoned or revised.

The use of these new processes resulted in a further 4744 participants being recruited in the following 10 months and resulted in the trial being completed ahead of schedule. The proportion of eligible participants joining the trial also increased from 33% to 57%.

#### Case study 3: the LIFELAX trial - use of the model as an audit tool

LIFELAX is a trial of diet and lifestyle vs laxatives in the management of constipation in older people [29]. From the outset, the trial team attempted to consider the trial in business terms. The coordinating team identified potential gains and benefits that would accrue from participation in the trial and conveyed messages tuned to the distinctive needs of target groups (nursing staff, administrators etc) through presentations and training materials. A marketing plan was developed and adopted, which included the trial's unique selling points. Recognising there would be no direct material benefit for primary care doctors (general practitioners) in taking part, the marketing strategy was targeted towards identifying all potential gains and benefits that would accrue from participating in the trial. Despite this approach, the trial failed to recruit to target. In response to this the LIFELAX coordinating team used the model as an audit tool to retrospectively assess the recruitment strategies adopted within the trial and to learn lessons for future trials.

Using the model, the trial team identified that they had failed in their early attempts to recruit public advocates for the trial. It also identified that patient representative activities were limited to involvement in the Trial Steering Committee (TSC) only and would have benefitted from wider involvement throughout the trial processes. The team also believed that if they had adopted techniques to increase awareness of the participating general practices this would have resulted in greater support and commitment, eg through regular publication of lists of participating practices.

Salient lessons from all three case studies are summarised in Table 2.

**Table 2:** Implementation of the business model

2.1: Case study one - using the model to develop trial processes		
Domains and Component	Issue Identified	Action Taken

1. Building brand values		

1a. Developing brand values	Identify the key positive qualities which collaborators associated with the successful delivery of the previous CRASH trial (i.e. the 'CRASH brand').	Conducted a survey of key collaborators from the previous trial to ascertain reasons for participation in that trial and what aspects of the coordinating centre's performance could be improved. Identified that continued use of the theme from the previous trial was useful to build on positive image already established.

1b. Gaining legitimacy and prestige	Need to enshrine the factors identified by the CRASH trial collaborators into CRASH-2.	Trial was coordinated by an academic institution, funding was by a non-commercial organisation and respected individuals identified and engagement maintained throughout CRASH-2.

1c. Signalling worthiness	The trial increased workload for clinical teams and the need to keep this to a minimum.	Simplified design and minimised procedures. Emphasized the values of adding to the clinical evidence and knowledge and prestige gained from individual participation (e.g. training in trials/authorship potential).

2. Product and market planning		

2a. Providing simple, complete processes	Recognised that, to maximise recruiting site participation and follow-up of participants, processes would have to be simple.	Minimised tasks that require to be done at recruiting sites and maximised those done by coordinating centre. Built in flexibility to accommodate varying local needs.

2b. Strategies to overcome resistance	Factors that may cause potential collaborator to reject or not fully commit to CRASH-2 were identified.	Coordinating centre created training responses and frequently asked questions were made available to collaborators to address issues (e.g. how to explain the trial, how to incentivise junior staff to recruit).

2c. Adopting an explicit marketing plan	Need for a marketing plan and to engage with a wide variety of people and organisations.	Developed a formal marketing plan including definition of groups that needed to buy into the trial.

3. Making the sale		

3a. Engaging active sponsors, champions and change agents	Need to recognise who the groups, networks and experts in the disease area are.	Engaged and maintained support of groups such as disease specific networks and patients and their representatives, including within participating countries to ensure any local sensitivities were addressed.

3b. Delivering a multi-audience, multi-level message	Different groups of people require the same information in varying formats to address concerns.	Ensured that when the same information was being presented to different groups (e.g. medical staff and nurses), the key messages were presented in ways that addressed all needs and concerns.

3c. Achieve buy-in (in public)	Recognition that the trial impacted on many more people at participating sites than the trial team and that a confirmation of commitment made publically by collaborators is more likely to result in their active participation.	Local collaborators were asked to raise awareness of the trial to anyone involved in the care of the trial participants. Collaborators were invited to state their commitment to the trial in public (e.g. at trial meetings).

4. Maintaining engagement		

4a. Ensuring positive 'moments of truth'	The need to be efficient, honest and timeous.	The central coordinating team tried to ensure that all collaborators had a clear and complete response to any queries. Any failures in procedures were reported honestly, with an apology and with a plan as to how to resolve any problems.

4b. Providing frequent positive reinforcement	Many local team members (e.g. doctors and nurses) were crucial contributors to ensuring the trial was successful at their site.	Recognised and rewarded acts (e.g. completing trial paperwork) by sending personalised texts, small tokens of appreciation and certificates of participation. Enabled networking and sharing of experiences.

4c. Facilitating incorporation into routines	As many trial procedures as possible should be incorporated into local routine.	Supported this through provision of additional helpful processes (e.g. providing trial labels to be attached to blood transfusion forms) to assist clinical staff to remember the trial as part of their standard care routine.

2.2: Case study two - using the model as a diagnostic tool		

Domains and Component	Issue Identified	Action Taken

1. Building brand values		

1a. Developing brand values	TXT2STOP set out to establish itself as a high quality trial as reflected by the manner in which it dealt with participants. Some participants hearing about the trial via adverts reported concerns about the trial being a scam.	Team amended the way the trial was presented in information to address this, paying particular focus to the potential benefit of the intervention to the NHS if proven effective and stating links to university.

1b. Gaining legitimacy and prestige	A need to clarify that the trial was funded and endorsed by a respected Institute.	The University links were stated in letters and other trial documents. The trial was funded by a non commercial institution.

1c. Signalling worthiness	There was a need to ensure that the general practitioners were not 'out of pocket' as a result of participating in the trial.	Appropriate National Health Service support costs were secured to support the general practitioners in order that they were not financially compromised as a result of participating in the trial. The importance of the trial question and the potential benefit of the intervention to the NHS, if proven effective were outlined in study information.

2. Product and market planning		

2a. Providing simple, complete processes	Workload at the sites had to be reduced as much as possible.	To facilitate recruitment and reduce the burden at recruiting general practices, letters were sent directly to smokers on the practice lists, thus negating the need for the doctors and nurses to tell their patients about the trial.

2b. Strategies to overcome resistance	It was necessary to identify what the benefits to each of the participating groups were.	The central team identified what would encourage the main participants (smokers, health care teams and smoking cessation services) to participate.

2c. Adopting an explicit marketing plan	Due to low recruitment figures during the early months of recruitment the marketing strategy had to be reviewed.	Adverts for the trial were evaluated and only effective adverts on effective media were repeated. We started to market the trial to potential participants who had registered interest but who had not gone on to either consent or not consent to trial inclusion.

3. Making the sale		

3a. Engaging active sponsors, champions and change agents	Clarity was required as to who the main gatekeepers were.	Relationships were established with key gate keepers such as the Scottish and English smoking cessation websites who promoted the trial.

3b. Delivering a multi-audience, multi-level message	Appropriate wording and means of communication are required.	Letters and text messages to potential participants were worded to focus on the different motivations that smokers may have for attempting to quit and joining a trial.

3c. Achieve buy-in (in public)	There was a need to use different media sources to indicate endorsement and promote the study.	The smoking cessation service research network distributed information about the trial to all participating services.

4. Maintaining engagement		

4a. Ensuring positive 'moments of truth'	Being allocated to the control group was a difficult experience for many participants.	We ensured that all research assistants involved in randomisation were trained in how to respond to participants' disappointment (and at time anger). It was acknowledged that being allocated to the control group was disappointing but reinforced that being involved in the trial would enable the intervention to be made publically available for everyone, if proven effective.

4b. Providing frequent positive reinforcement	Information provision requires not only to be regular but effective.	The effectiveness of all promotions were monitored and evaluated. Only effective strategies were repeated.

4c. Facilitating incorporation into routines	Clarity and ease of procedures were required.	All new processes were incorporated into standard operating procedures.

2.3: Case study three - using the model as an audit tool		

Domains and Component	Issue Identified	Action Taken

1. Building brand values		

1a. Developing brand values	Recognition that the brand values are vital in clarifying what the trial will deliver (to health care professionals' patients etc).	Continue to use distinct messaging and appropriately qualified professionals to develop trial brand.

1b. Gaining legitimacy and prestige	Recognise the key and appropriate individuals and institutions.	Ensure that it is obvious who the trial is associated with.

1c. Signalling worthiness	Ensured that any monetary costs to clinical investigators was reimbursed and provided training that would be generalisable in the trial setting and beyond.	Roll out any training and information to all participating clinicians at the end of trial so that the 'value' will be, eventually, available to all.

2. Product and market planning		

2a. Providing simple, complete processes	Assistance required with electronic queries to search for patients.	Provide assistance with any issues and consider reviewing protocol if necessary to amend and simplify processes.

2b. Strategies to overcome resistance	Initial assumption that resistance to participation would be low but barriers to participation were subsequently identified.	Be open minded about resistance and devise strategies in advance to overcome.

2c. Adopting an explicit marketing plan	There is a need for a formal marketing plan.	Identified trial's unique selling points for each of the categories in order to highlight benefits of involvement and supporting study.

3. Making the sale		

3a. Engaging active sponsors, champions and change agents	Public advocates and patient representatives require to be on board the study from the outset and across a wide range of activities.	Team had failed in early attempts to recruit public advocates (GP champions, PCT R&D Staff) and it would have been beneficial if patient representative activities had been more extensive than only participation in the Trial Steering Committee.

3b. Delivering a multi-audience, multi-level message	One size does not fit all with regard to communicating and addressing motivation of different target groups.	Presentations (contents and style) were tailored to fit with the target audience (e.g. family doctors, nurses, practice managers).

3c. Achieve buy-in (in public)	Collaborators who make a silent decision to commit to the study forgot easily - it is necessary to get a senior person within the institution to announce their participation and support of the study to the rest of their team.	Collated anecdotal evidence that the lack of awareness in general practices suggested that team support would have been greater had the senior management announced involvement. Also realised that techniques such as publishing lists of signed up practices would have assisted in signalling the commitment of those sites to the study.

4. Maintaining engagement		

4a. Ensuring positive 'moments of truth'	A clear and efficient system of handling queries is required in order to ensure an accurate and timely response.	The team set up a dedicated 'LIFELAX.queries@......' email address, which was checked routinely and messages passed to the most appropriate team member for response.

4b. Providing frequent positive reinforcement	There is a need to undertake training, including follow-up training, at all sites.	The study team largely achieved this component, the result being that sites which underwent training and became active recruiting sites remained committed to the trial until the end.

4c. Facilitating incorporation into routines	This aspect of the study was fairly difficult for LIFELAX as the patient search was a one-off.	The central team attempted to demonstrate to the staff at the participating general practice sites that the principles and techniques of behaviour change taught in LIFELAX could be incorporated into routine practice and should not be seen as study specific training only.

## Discussion

It is crucially important for a trial to recruit to its target sample size. Poor recruitment can lead to an underpowered study, which may result in clinically important effects being missed (i.e. judged to be non-significant) [30]. A non-significant finding increases the risk that an effective intervention will be abandoned before its true value is established, or that there will be a delay in demonstrating this value while more trials or meta-analyses are done. Underpowered trials also raise an ethical problem: trialists have exposed participants to an intervention with uncertain benefit but may still be unable to determine whether an intervention does more good than harm on completion of the trial.

Often trialists apply for recruitment extensions to achieve the desired sample size. This, however, invariably adds to the cost of running trials and is likely to result in delays to the results from these ongoing trials being available to inform clinical practice. A further consequence is that if more resources are diverted to these "failing" trials, there is less money available to support new trials, which may result in less research being undertaken [17].

The two reviews summarised in the paper highlight the potential benefits of interventions such as the use of opt-out, rather than opt-in, procedures for contacting potential trial participants and greater use of open designs where participants know which treatment they are receiving in the trial. However, some of these strategies have disadvantages, which may limit their widespread use. For example, the use of opt-out procedures remains controversial and the ethics of this approach has been challenged [31]. The use of open designs may also reduce the scientific rigour of the trial as, by definition, blinding is not adopted. However using an active comparator rather than placebo may, on the other hand, offer both recruitment and applicability advantages [3,16].

In addition, evidence from the case studies presented in this paper suggests that a marketing approach is feasible in trials. In the TXT2STOP trial all the new interventions and processes adopted fitted into the marketing framework (tool) and a marked improvement in recruitment followed. On first appraisal, there is an apparent clash of values between business and medicine - among key business values are profit and competition, while among the traditional values of health care researchers are service, advocacy, and altruism. However, they share common goals; businesses strive to find customers and encourage them to buy what is on offer, clinical trials strive to find doctors and patients/volunteers and encourage them to sign up and stay on board. One reason that appears to contribute to an unwillingness to use marketing concepts within clinical trials is that trialists are unfamiliar with the business language and are unsure how to translate these concepts into recognisable trial procedures (personal communication). In an attempt to address this, we have presented example 'translations' of the key concepts in Table 1.

It is also clear from the case studies that the terms in the model have to be operationalised individually for each trial. Market segments will be different for each trial, as will the range of stakeholders. This, therefore, requires extensive planning ahead of starting recruitment within the trial, and this planning phase should be explicitly accommodated within the timetable for the trial. It is not clear how this planning phase might be funded, although it is possible that this should be included in the trial grant application.

## Strengths and weaknesses

This is the first paper to our knowledge which has presented some empirical evidence on the implementation of the business model. This is a strength of the paper and provides useful evidence on the feasibility and acceptability of the model to the trial community. However, our case studies were not selected randomly - all were known to the authors as having attempted to implement the model.

From our case studies it is not yet clear which elements of marketing strategies are most effective or whether it is the adoption of a whole approach that is important. It is also not known if the improvement in recruitment would have occurred had 'marketing strategies' not been adopted. For example, in TXT2STOP many of the strategies to improve recruitment had been initiated prior to referring to the marketing model (although it was noted that all activities undertaken fitted into the marketing framework). In addition, some of the case studies we have presented that have used the business model could be considered atypical as one study involved healthy volunteers, while two other studies were led by a researcher who was very familiar with the development and process of the business model. Investigators using the model report finding it a useful framework for conceptualising recruitment and for identifying where further interventions or changes in trial process should be considered. The case studies suggest the model can be a useful tool and that there may be a link between the use of the model and increased recruitment.

## Further research required

The use of the business model requires further empirical evaluation. Further work is also required to develop a manual or similar tool to operationalise the model consistently from trial to trial. Further research into how effective the model would be as a disaster recovery tool would also enhance the evidence. If however, the findings of our case studies are replicated in other arenas, it is likely that we could conclude that use of the model increases recruitment to trials.

## Conclusion

Recruitment is a challenge for most trials, sometimes a very significant one. Trialists use a variety of approaches to support recruitment but empirical evidence on what works, when and why is rather scarce. The business model is a new approach to trial management and offers something that has been lacking: a consistent framework for planning and managing recruitment. The model seems promising based on results from a small number of case studies. What is needed now are more examples of its use. If the early promise of the model was replicated in other trials, especially those of trialists uninvolved in the development of the model, this would represent a major advance in the conduct and management of clinical trials.

## Competing interests

The authors declare that they have no competing interests.

## Authors' contributions

AMM wrote the first draft of the paper and co-ordinated and contributed to the final version. ST and MKC contributed to the paper. HS contributed to the paper, in particular to the content of case study one. CF and RK contributed to the paper, in particular to the content of case study two. CS contributed to case study three in particular. All authors read and approved the final manuscript.
